# Congenital cytomegalovirus related intestinal malrotation: a case report

**DOI:** 10.1186/s13052-016-0318-8

**Published:** 2016-12-07

**Authors:** Claudia Colomba, Mario Giuffrè, Simona La Placa, Antonio Cascio, Marcello Trizzino, Simona De Grazia, Giovanni Corsello

**Affiliations:** Department of Sciences for Health Promotion and Mother-Child Care “G.D’Alessandro”, Via del Vespro 129, 90127 Palermo, Italy

**Keywords:** Cytomegalovirus, Malrotation, Volvulus, Congenital

## Abstract

**Background:**

Cytomegalovirus is the most common cause of congenital infection in the developed countries. Gastrointestinal involvement has been extensively described in both adult and paediatric immunocompromised patients but it is infrequent in congenital or perinatal CMV infection.

**Case presentation:**

We report on a case of coexistent congenital Cytomegalovirus infection with intestinal malrotation and positive intestinal Cytomegalovirus biopsy. At birth the neonate showed clinical and radiological evidence of intestinal obstruction. Meconium passed only after evacuative nursing procedures; stooling pattern was irregular; gastric residuals were bile-stained. Laparatomy revealed a complete intestinal malrotation and contextually gastrointestinal biopsy samples of the appendix confirmed the diagnosis of CMV gastrointestinal disease. Intravenous ganciclovir was initiated for 2 weeks, followed by oral valgancyclovir for 6 month.

**Conclusion:**

CMV-induced proinflammatory process may be responsible of the interruption of the normal development of the gut or could in turn lead to a disruption in the normal development of the gut potentiating the mechanism causing malrotation. We suggest the hypothesis that an inflammatory process induced by CMV congenital infection may be responsible, in the early gestation, of the intestinal end-organ disease, as the intestinal malrotation. CMV infection should always be excluded in full-term infants presenting with colonic stricture or malrotation.

## Background

Cytomegalovirus (CMV) is the most common cause of congenital infection in the developed countries, affecting 0.1–2% of live born infants. The majority of children with congenital CMV infection (approximately 85%–90%) do not have clinical findings at birth [1].

Both maternal primary and recurrent infection during pregnancy can result in congenital infection of the infant. It has been thought that children born to mothers with primary CMV infection during pregnancy are much more likely to have symptoms at birth and suffer sequelae than those born congenitally infected from a maternal recurrent CMV infection [1, 2]. More recent data have shown the severity of newborn disease do not differ between primary and non primary infection groups [3].

The most commonly observed physical findings are petechial rash, jaundice and hepatosplenomegaly with neurologic abnormalities such as microcephaly and lethargy. Ophthalmologic examination reveals chorioretinitis and/or optic atrophy in approximately 10% of symptomatic infants and sensorineural hearing loss is detected in more than two-thirds of symptomatic newborns [1].

Gastrointestinal involvement due to CMV infection has been extensively described in both adult and paediatric immunocompromised patients and transplant recipient but it is rare in immunocompetent adult. The entire GI tract may be involved, with the esophagus and colon being the most common sites. The clinical spectrum ranges from relatively minor symptoms such as diarrhea, mouth ulcers, esophagitis and malabsorption to more serious symptoms such as hemorrhage, obstruction and perforation. [4–6]. However, CMV in association with ulcerative colitis has been recognized for 50 years [7].

Gastrointestinal involvement in congenital or perinatal CMV infection is infrequent [8, 9].

We report, for the first time, on a case of coexistent congenital CMV infection with intestinal malrotation and positive intestinal CMV biopsy.

## Case presentation

A male neonate was born by vaginal delivery at 39^+3^ weeks of gestation to a 30-year-old gravida 2 and para 2 mother. The newborn had a birth weight of 3260 g (30th percentile), a length of 51 cm (59th percentile) and a head circumference of 34 cm (25th percentile). Apgar scores were 9 and 10 at 1 and 5 min, respectively. Clinical examination at birth was normal, abdomen was palpable and the rectum was permeable. Mother’s prenatal routine TORCH serology screens were negative for HIV, hepatitis B and C, VDRL/TPHA; negative IgM antibodies and positive IgG antibodies for Rubella, Toxoplasma and CMV were detected in the first trimester. Further investigations up to 34 weeks revealed a reactivation for CMV (both positive IgG and IgM antibodies) with a high CMV IgG avidity, speaking for a maternal secondary CMV infection.

Prenatal ultrasound scannings at 34 and 37 weeks of gestation revealed dilatation of the small intestine. A fetal echocardiography at 21st week gestation showed a structurally normal heart.

At birth the neonate showed clinical and radiological evidence of intestinal obstruction: meconium passed only after evacuative nursing procedures; stooling pattern was irregular; gastric residuals were bile-stained; radiological bowel gas pattern and barium enema were consistent with intestinal obstruction. The blood investigations revealed mildly raised C-reactive protein levels (1,2 mg/dL; normal value <0,5). Haemoglobin, white cell count, platelets, liver enzymes were within normal limits. Blood and urine cultures for bacteria were negative. Urine was screened for cytomegalovirus on day one confirming congenital CMV infection (>2.700.000 copies/ml). CMV DNA was detected (6343 copies/ml) by Polymerase Chain Reaction (PCR) in blood sample on day ten. Patient was managed with nasogastric decompression; enteral feeding was discontinued. Laparatomy performed on day 10 revealed a complete intestinal malrotation and contextually gastrointestinal biopsy samples of the appendix confirmed the diagnosis of CMV gastrointestinal disease. Actually, PCR on tissue samples revealed 1096 CMV-genome copies/mg. Postoperative management was regular and the neonate made a good recovery. Serial abdomen and head ultrasound examination showed no evidence of hepatosplenomegaly or ventriculomegaly, except for echogenic periventricular foci next to the third ventricle consistent with calcification.

Intravenous ganciclovir was initiated on day 17 at a dose of 6 mg/kg twice daily for 2 weeks, followed by oral valgancyclovir at a dose of 16 mg/kg twice daily for 6 month.

Follow-up included fortnightly viral load, liver enzymes, creatinine and blood urea nitrogen monitoring. Urine viral load fell from >27 million copies/ml on day one to 121,000 copies/ml on day 37. Blood viral load was 6343 copies per millilitre on day 10, and, after 22 days of antiviral therapy, was no more detected. Serology for CMV showed positive IgG and negative IgM antibodies. Biochemistry was normal. No adverse effects from the treatment were seen and the baby remained asymptomatic. Neurological status remained normal. Serial ophthalmologic examination ruled out CMV chorioretinitis. Magnetic resonance imaging (MRI) at 8 weeks of age showed thinned corpus callosum and enlarged cisterna magna. Visual and hearing screenings with evocative visual potentials and otoacustic emissions were negative. At 2 month the infant showed good thriving, weighing 4800 g and with a head circumference of 37 cm. Neurologic examination was normal for age.

## Discussion

Malrotation is the generic term used to describe the consequences of an arrest of embryologic development of the midgut that can occur at any phase of pregnancy with variable consequences. Rotational anomalies, which may or may not be symptomatic, are estimated to occur in between 1 in 200 and 1 in 500 live births. Symptomatic malrotation is estimated to occur in 1 in 6000 live births. From the pathogenetic point of view the anatomical development of the intestinal tract is a complex process. Simply defined, malrotation is a failure during development of normal rotation of any part of the embryonic gut [10].

The classic presentation of malrotation with volvulus is thus that of a neonate with bilious vomiting. In our patient findings suggestive of bowel obstruction have been noted on prenatal sonography and clinical and radiological evidence of intestinal obstruction were present at birth. The majority of children with malrotation do not have any predisposing syndrome or genetic susceptibility. In our patient the intestinal malrotation was associated with congenital CMV infection and positive intestinal CMV biopsy. We think that in our case congenital CMV infection may be etiologically associated with rotational anomalies.

It is not clear the exact role of the CMV infection in the pathogenesis of intestinal malrotation.

CMV-induced proinflammatory process may be responsible of the interruption of the normal development of the gut or could in turn lead to a disruption in the normal development of the gut potentiating the mechanism causing malrotation.

On the other hands other authors described CMV as a viral agent that may be involved in surgical pathology in neonates [11]. To confirm the causal relationship between CMV and these pathologic findings, a large-scale longitudinal prospective screening of CMV in neonates with intestinal conditions should be performed.

## Conclusion

We suggest the hypothesis that an inflammatory process induced by CMV congenital infection may be responsible, in the early gestation, of the intestinal end-organ disease, as the intestinal malrotation. CMV infection should always be excluded in full-term infants presenting with colonic stricture or malrotation.

## Abbreviations

CMV: Cytomegalovirus
MRI: Magnetic resonance imaging
PCR: Polymerase Chain Reaction
